# The MDM2-inhibitor Nutlin-3 synergizes with cisplatin to induce p53 dependent tumor cell apoptosis in non-small cell lung cancer

**DOI:** 10.18632/oncotarget.4433

**Published:** 2015-06-10

**Authors:** Christophe Deben, An Wouters, Ken Op de Beeck, Jolien van Den Bossche, Julie Jacobs, Karen Zwaenepoel, Marc Peeters, Jan Van Meerbeeck, Filip Lardon, Christian Rolfo, Vanessa Deschoolmeester, Patrick Pauwels

**Affiliations:** ^1^ Center for Oncological Research (CORE), University of Antwerp, Antwerp, Belgium; ^2^ Department of Pathology, Antwerp University Hospital, Antwerp, Belgium; ^3^ Department of Medical Oncology, Antwerp University Hospital, Antwerp, Belgium; ^4^ Center for Medical Genetics, Department of Biomedical Sciences, University of Antwerp, Antwerp, Belgium; ^5^ Department of Thoracic Oncology, Antwerp University Hospital, Antwerp, Belgium; ^6^ Phase-1 Early Clinical Trials Unit, Antwerp University Hospital, Antwerp, Belgium

**Keywords:** cisplatin, MDM2, p53, NSCLC, synergism

## Abstract

The p53/MDM2 interaction has been a well-studied target for new drug design leading to the development of the small molecule inhibitor Nutlin-3. Our objectives were to combine Nutlin-3 with cisplatin (CDDP), a well-known activator of the p53 pathway, in a series of non-small cell lung cancer cell lines in order to increase the cytotoxic response to CDDP. We report that sequential treatment (CDDP followed by Nutlin-3), but not simultaneous treatment, resulted in strong synergism. Combination treatment induced p53's transcriptional activity, resulting in increased mRNA and protein levels of MDM2, p21, PUMA and BAX. In addition we report the induction of a strong p53 dependent apoptotic response and induction of G2/M cell cycle arrest. The strongest synergistic effect was observed at low doses of both CDDP and Nutlin-3, which could result in fewer (off-target) side effects while maintaining a strong cytotoxic effect. Our results indicate a promising preclinical potential, emphasizing the importance of the applied treatment scheme and the presence of wild type p53 for the combination of CDDP and Nutlin-3.

## INTRODUCTION

In recent years, cancer therapy has evolved from general treatment strategies to more specific targeted therapies, based on the genetic profile of individual tumors. Important progress has been made in understanding the underlying molecular mechanisms that drive tumorigenesis. These new findings have led to the discovery of new therapeutic targets, and consequently the development of new-targeted therapeutics. These tailor-made treatment modalities might improve the efficiency of cancer treatment, reduce common side effects by avoiding unnecessary toxicity and improve general outcome. Preclinical studies have become increasingly important in this setting. They allow to determine new combination strategies of these targeted agents with conventional chemo- and/or radiotherapeutics, but also to unravel the underlying molecular mechanisms and to define the optimal treatment schemes.

A well-know mechanism driving tumor formation is the disruption of the tumor suppressor protein p53. The protein plays an important role in the response to a variety of cellular stress signals by the induction of cell cycle arrest, senescence or apoptosis. The p53 pathway is disturbed in most cancers either by inactivating mutations, which occur in approximately 50% of all tumors, or by other mechanisms, suppressing p53 levels in the cancer cell. This makes the altered p53 pathway an attractive target for novel cancer therapies [1-5].

An interesting strategy is to target the interaction between p53 and its main negative regulator, the ‘murine double minute-2′ (MDM2) protein. MDM2 is part of a negative feedback loop in which p53 acts as a transcription factor for MDM2, that in turn acts as an E3 ubiquitin ligase targeting p53 for proteasomal degradation. Overexpression of MDM2 interrupts the well-controlled balance between p53 and MDM2, leading to malignant transformation of the cell. Increased MDM2 levels can result from *MDM2* gene amplification, which is assumed to occur in 10% of human tumors [6], or from the presence of a single nucleotide polymorphism (SNP309) in the promoter region of the *MDM2* gene [7]. Inhibiting the interaction between p53 and MDM2 might therefore restore the normal p53 function. In 2004, Vassilev et al. identified Nutlin-3, a small molecule inhibitor of the MDM2-p53 interaction with *in vitro* and *in vivo* antitumor activity [8]. The molecule is able to induce the activation of p53 downstream targets, cell cycle arrest and apoptosis [9]. Cancer cells with *MDM2* gene amplification were most sensitive to Nutlin-3 *in vitro* and *in vivo*, but Nutlin-3 also showed good efficacy against tumors with normal MDM2 expression. This shows that a wide array of patients with wild type p53 could benefit from the treatment with antagonists of the p53-MDM2 interaction [4].

**Figure 1:**
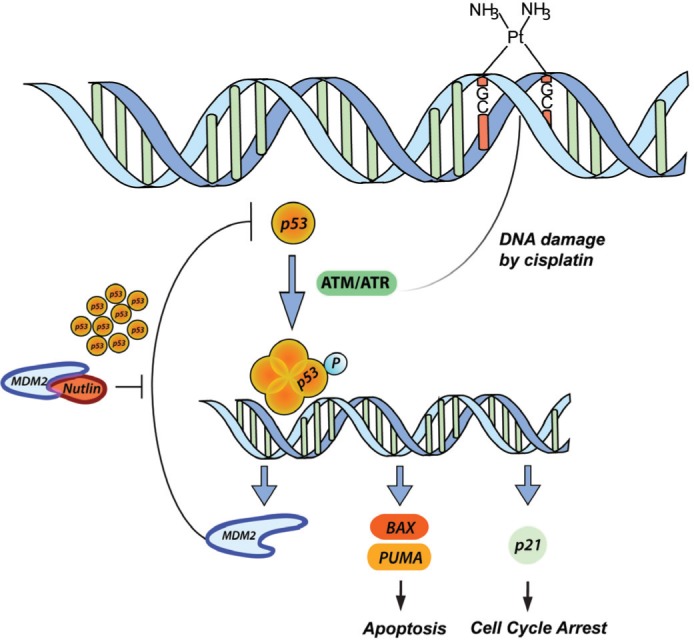
p53 pathway in response to CDDP and Nutlin-3 therapy CDDP induces DNA damage by forming DNA cross-links, thereby inducing the activation of ATM/ATR. The latter are able to activate p53 by phosphorylation and the formation of a p53 tetramer, which acts as a transcription factor for among others MDM2 (negative regulation), BAX and PUMA (apoptosis) and p21 (cell cycle arrest). The inhibition of MDM2 by Nutlin-3 results in a high increase in p53 levels in response to CDDP treatment resulting in a synergistic cytotoxic effect.

Although Nutlin-3 shows a good efficiency as a single agent, the anti-tumoral effect might be enhanced when it is administered in combination with DNA-damaging agents in p53 wild type tumors. In this study, we focused on the combination of Nutlin-3 with CDDP (*cis*-diamminedichloroplatinum(II); cisplatin), a well-known activator of the p53 pathway, in a series of non-small cell lung cancer (NSCLC) cell lines with different p53 background (Figure 1). We selected the commonly used A549 cell line based on its genotype (p53 wild type, EGFR/ALK/ROS1 negative) and suitability as transduction host, for which we used a vector containing anti-p53 specific shRNA. In addition, CRL-5908 was used, harboring the R273H p53 hotspot mutation, resulting in a conformational change in the p53 protein and inhibiting its transcriptional activity. Currently, CDDP treatment is used in platinum doublet therapy for the treatment of late stage EGFR/ALK/ROS1 negative tumors. However, tumor cells lacking functional p53 are prone to be more resistant to CDDP treatment [10]. Increasing functional p53 levels by Nutlin-3 could enhance the cytotoxic effect of CDDP. This combination regimen has been shown to be beneficial in both sarcoma cell lines and CDDP resistant ovarian cancer cell lines [9, 11]. On the other hand, Nutlin-3 is able to protect both normal and tumor wild type p53 cells from mitotic inhibitors like paclitaxel by inducing G1 and G2 phase arrest [12-15]. Therefore, we studied the effect of the treatment schedule for the combination of Nutlin-3 with CDDP by administrating these drugs either simultaneously or sequentially (CDDP followed by Nutlin-3). Prior treatment with Nutlin-3 would activate p53 in a non-genotoxic way resulting in cell cycle arrest rather than apoptosis; extending time for DNA repair mechanisms to take place in response to CDDP induced DNA damage before progression through critical phases of the cell cycle [16, 17]. Therefore, prior treatment with Nutlin-3 was not tested in this study. As regions with reduced oxygen levels often characterize tumors, a part of the study was performed under both normoxic and hypoxic (<0.1% O_2_) conditions.

## RESULTS

### The role of wild type p53 in the response to Nutlin-3 monotherapy

**Figure 2:**
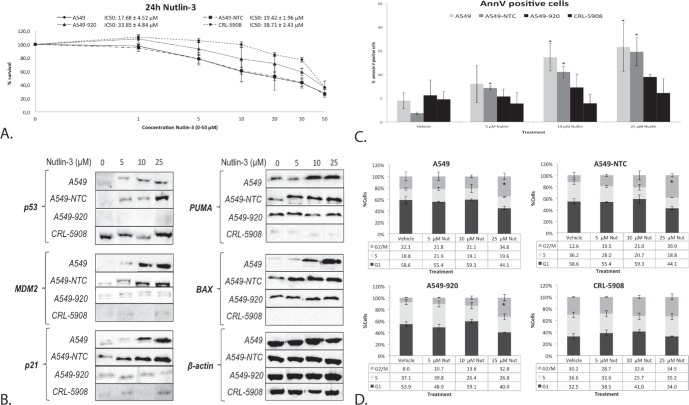
The response to Nutlin-3 monotherapy was strongest in the presence of wild type p53 **A.** Survival curve after 24 hours of treatment with Nutlin-3 (0-50 μM) in the p53 wild type cell lines A549 and A549-NTC, the p53 deficient cell line A549-920 and p53 mutant cell line CRL-5908. The corresponding IC_50_-values are presented as mean ± SD in the figure. **B.** Protein expression levels of p53 and its main transcription targets MDM2, p21, PUMA, and BAX after treatment with 0, 5, 10 or 25 μM Nutlin-3 in all cell lines. **C.** Percentage of Annexin V PerCP positive cells after 0, 5, 10 or 25 μM Nutlin-3 in all cell lines. **D.** Cell cycle distribution after Nutlin-3 monotherapy, Cells were stained with Propidium Iodide and DNA content was measured by flowcytometric analysis. Cells were divided in 3 groups: G1 phase (2n); S-phase (2n-4n); and G2/M phase (4n). (*p < 0.05: significant difference compared to vehicle treated sample).

To determine the role of the p53 status in the cytotoxic effect of Nutlin-3, cells with a different p53 background were treated with 0-50 μM Nutlin-3 for 24 hours. The p53 wild type cell line A549 and its non-template control A549-NTC were clearly more sensitive to Nutlin-3 (IC_50_: 17.68 ± 4.52 μM and 19.42 ± 1.96 μM, respectively), with an IC_50_ value significantly lower than the isogenic p53 deficient cell line A549-920 (33.85 ± 4.84 μM; *p*-value: 0.002) and p53 mutant cell line CRL-5908 (38.71 ± 2.43 μM; *p*-value < 0.001) (Figure 2A). To obtain a better insight in the underlying mechanisms, all cells were treated with 5 μM, 10 μM or 25 μM Nutlin-3 (corresponding with the IC_20_, IC_40_ and IC_60_ value in the p53 wild type cell line A549) and p53 expression levels were assessed. In contrast to the p53 deficient or mutant cell lines, increasing p53 protein levels were observed in accordance with increasing levels of Nutlin-3 in the p53 wild type cell lines (Figure 2B). Lower levels of p53 and p21 were observed for CRL-5908 treated with 10 μM Nutlin-3 due to a lower concentration of protein loaded, corresponding with β-actin control levels. Nutlin-3 treatment led to the activation of wild type p53, resulting in increased protein levels of its main transcription targets PUMA, BAX, p21 and MDM2 (Figure 2B), which in turn led to a significant increase in annexin V positive cells (Figure 2C) in the p53 wild type cell lines, but not in the p53 deficient and mutant cell lines. A significant G2/M phase arrest was observed in A549 and A549-NTC at 25 μM Nutlin-3 treatment, but also in the p53 deficient cell line A549-920, due to the presence of residual p53 and p21 protein. The p53 mutant cell line did not show any significant change in G2/M phase arrest (Figure 2D).

### Nutlin-3 strongly synergizes with CDDP after sequential combination therapy

#### Cell survival and synergism

**Figure 3:**
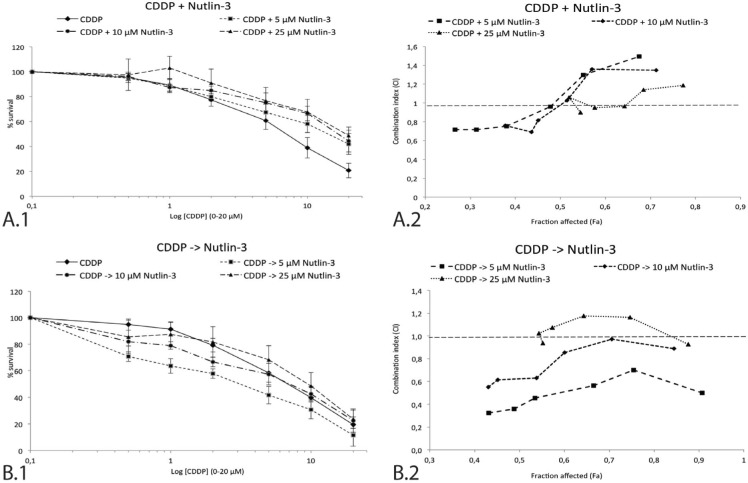
Survival curve and combination index (CI) of the sequential and simultaneous combination therapy in the p53 wild type cell line A549 **A.** 1. Survival curve after 24 hours of CDDP (0-20 μM) monotherapy and in simultaneous combination with 5 μM, 10 μM, or 25 μM Nutlin-3. 2. The corresponding combination index for each Nutlin-3 concentration is shown in detail on the right. Each data point represents the corresponding CDDP concentration (0.5-1-2-5-10-20 μM). **B.** 1. Survival curve after 24 hours of CDDP (0-20 μM) monotherapy and sequential combination therapy with 5 μM, 10 μM, or 25 μM Nutlin-3. 2. The corresponding combination index for each Nutlin-3 concentration is shown in detail on the right. Each data point represents the corresponding CDDP concentration (0.5-1-2-5-10-20 μM). The supporting data for this figure (Mean IC_50_-values and mean CI) can be found in Table 1.

To investigate the potential interaction between Nutlin-3 and CDDP in the p53 wild type NSCLC cell line A549, tumor cells were incubated with 0-20 μM CDDP combined with either simultaneous or sequential treatment of 0 μM, 5 μM, 10 μM or 25 μM Nutlin-3 for 24 hours. A clear difference was observed between the two treatment schemes, supported by the data in Table 1 and Figure 3. After sequential treatment, the strongest synergistic effect was observed in the lowest concentrations ranges of both Nutlin-3 and CDDP (<CI> = 0.486 for CDDP -> 5 μM Nutlin-3) (Figure 3B), resulting in a significant reduction in CDDP IC_50_-value (6.28 ± 1.62 *vs*. 2.52 ± 0.57 μM, *p*-value = 0.003). On the contrary, Nutlin-3 seemed to protect cells from the cytotoxic effect of medium to high concentrations of CDDP when administrated simultaneously, resulting in an antagonistic effect at higher concentrations of CDDP. However, a weak synergistic effect at low concentrations of both Nutlin-3 and CDDP (<CI> = 0.990 for CDDP + 5 μM Nutlin-3) was found (Figure 3A).

The induction of a hypoxic environment led to a noticeable decrease in CDDP IC_50_-value when sequentially combined with 5 μM Nutlin-3, although not significant (6.73 ± 0.30 *vs*. 4.69 ± 0.85 μM, *p*-value = 0.100). In this hypoxic environment, sequential therapy induced a synergistic effect, yet slightly weaker than the synergism observed under normoxic conditions (<CI> = 0.625 *vs*. <CI> = 0.486). As hypoxic conditions did not inhibit the synergistic effect we conducted the following experiments under normal oxygen levels.

**Table 1:** Cytotoxicity and synergism of the CDDP and Nutlin-3 combination therapy in the p53 wild type cell line A549

Cytotoxicity and synergism					
Treatment	Normoxia (0-20 μM CDDP)				
	IC_50_	StDev	*p*-value*	CI	StDev
24 h CDDP	5.51	0.66	/	/	/
24 h CDDP -> 5 μM Nutlin-3	2.67	0.26	0.003	0.486	0.138
24 h CDDP -> 10 μM Nutlin-3	5.46	0.37	0.788	0.752	0.174
24 h CDDP -> 25 μM Nutlin-3	9.13	2.70	0.003	1.050	0.108
24 h CDDP	6.35	2.30	/	/	/
24 h CDDP + 5 μM Nutlin-3	15.36	3.93	0.008	0.990	0.333
24 h CDDP + 10 μM Nutlin-3	22.39	7.63	0.008	1.000	0.296
24 h CDDP + 25 μM Nutlin-3	16.29	3.26	0.016	1.033	0.114
**Treatment**	**Hypoxia (0-20 μM CDDP)**				
	**IC_50_**	**StDev**	*p*-value*	**CI**	**StDev**
24 h CDDP	6.73	0.30	/	/	/
24 h CDDP -> 5 μM Nutlin-3	4.68	0.85	0.100	0.625	0.082
24 h CDDP -> 10 μM Nutlin-3	5.72	0.77	0.200	0.792	0.116
24 h CDDP -> 25 μM Nutlin-3	6.62	1.46	0.629	0.975	0.211
24 h CDDP	6.29	0.89	/	/	
24 h CDDP + 5 μM Nutlin-3	11.24	1.63	0.057	1.068	0.361
24 h CDDP + 10 μM Nutlin-3	15.86	5.59	0.029	1.076	0.330
24 h CDDP + 25 μM Nutlin-3	11.30	1.48	0.057	1.227	0.113

The table gives an overview of the IC_50_-value of CDDP after both monotherapy and simultaneous/sequential combination therapy with Nutlin-3 under normoxic or hypoxic conditions. The average combination index (CI) is provided for each combination therapy. CI > 1 indicates an antagonistic effect, CI = 1 an additive effect and CI < 1 a synergistic effect.

* (*p* < 0.05: significant difference in IC_50_-value compared to CDDP monotherapy)

#### Activation of wild type p53

**Figure 4:**
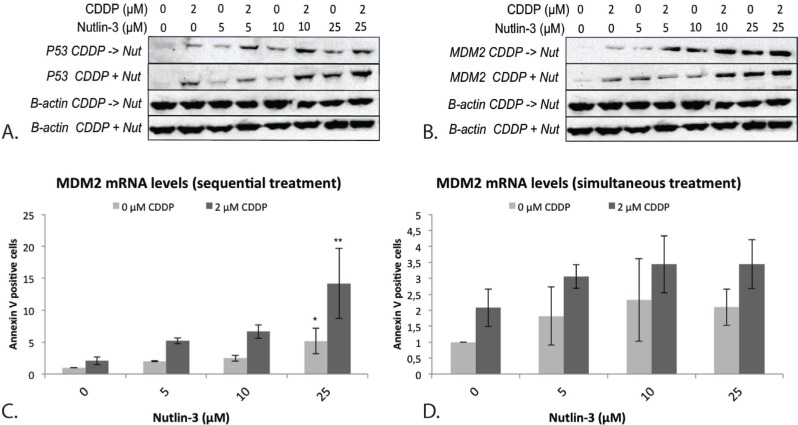
Expression of the p53 protein and its negative regulator MDM2 after simultaneous and sequential combination therapy in the p53 wild type cell line A549 **A.** p53 protein levels after treatment **B.** MDM2 protein levels after treatment; β-actin was used as an internal standard. **C.** MDM2 mRNA levels after sequential treatment. **D.** MDM2 mRNA levels after simultaneous treatment. (*p < 0.05: significant difference compared to 0 μM CDDP; **p < 0.05: significant difference compared to 2 μM CDDP).

The p53 protein levels strongly increased after sequential combination therapy, even at a low dose of Nutlin-3, compared to CDDP and Nutlin-3 monotherapy (Figure 4A). After simultaneous treatment this effect was only observed at higher concentrations of Nutlin-3.

Next, the activation status of p53 was determined by determining the mRNA and protein levels of its main transcription targets MDM2, PUMA, BAX, and p21 as well as their downstream effects, namely apoptosis (PUMA and BAX) and cell cycle arrest (p21).

#### Negative regulation: induction of MDM2

The mRNA levels of MDM2 were markedly increased after sequential treatment, but not after simultaneous treatment, compared to both CDDP and Nutlin-3 monotherapy (Figure 4C) although a statistically significant increase was only observed when CDDP was combined sequentially with 25 μM Nutlin-3 (Figure 4C, Figure 4D). At the MDM2 protein level, a noticeable increase was seen earlier with a lower dose of Nutlin-3 (5 μM) for the sequential treatment compared to simultaneous treatment. At higher doses of Nutlin-3, no difference was seen between sequential and simultaneous treatment (Figure 4B).

#### Induction of apoptosis

**Figure 5:**
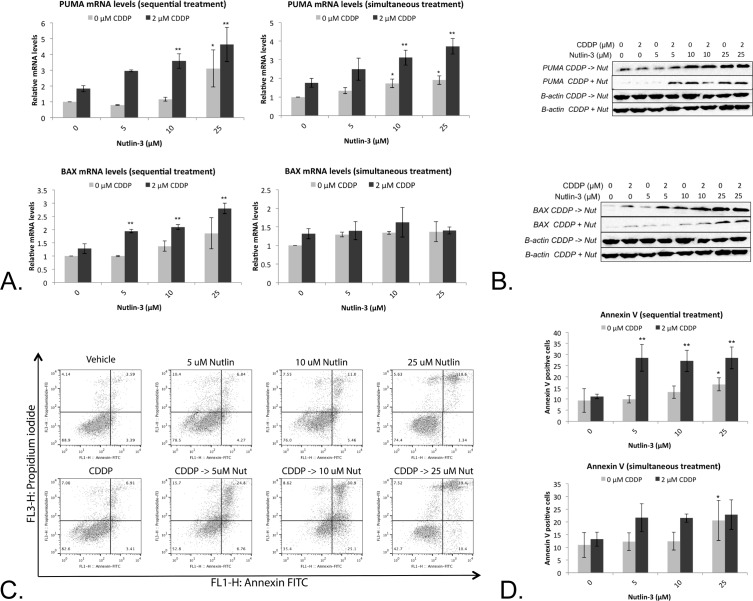
Nutlin-3 enhanced the apoptotic effect of CDDP in the p53 wild type cell line A549 **A.** Relative mRNA expression levels of p53's main apoptotic targets PUMA and BAX. Cells were treated with either 2 μM CDDP; 5 μM, 10 μM or 25 μM Nutlin, or a sequential (CDDP -> Nutlin)/simultaneous combination therapy of both drugs for 24 hours. **B.** Corresponding protein levels of PUMA and BAX, β-actin was used as internal standard. **C.** Cells were labeled with Annexin V-FITC (AnnV) and Propidium Iodide (PI) and measured by flowcytometric analysis. Dot-plot: LL = AnnV-/PI-; LR = AnnV+/PI-; UR = AnnV+/PI+; UL = AnnV-/PI+. **D.** Percentage of Annexin V FITC positive cells. (*p < 0.05: significant difference compared to 0 μM CDDP; **p < 0.05: significant difference compared to 2 μM CDDP).

Treatment with Nutlin-3 led to a moderate (5 μM and 10 μM) to significant (25 μM) increase in PUMA mRNA levels. A similar effect was observed after simultaneous and sequential combination therapy, for which a significant effect was observed after treatment with 10 and 25 μM Nutlin-3 in combination with 2 μM CDDP (Figure 5A). This effect was translated to the PUMA protein levels (Figure 5B). Despite these similar effects after simultaneous and sequential therapy, a significant increase in BAX mRNA levels was observed only after sequential combination therapy, but not after monotherapy or simultaneous combination therapy (Figure 5A). Again, this was also reflected at the BAX protein levels (Figure 5B).

As both PUMA and BAX are able to induce p53 dependent apoptosis, the percentage of Annexin V positive cells and PI positive cells was determined (Figure 5C). Sequential treatment led to a significant increase in Annexin V positive cells even at low concentration of Nutlin-3 (5 μM), which was not observed after simultaneous or CDDP/Nutlin-3 monotherapy treatment (Figure 5D).

#### Cell cycle distribution

**Figure 6:**
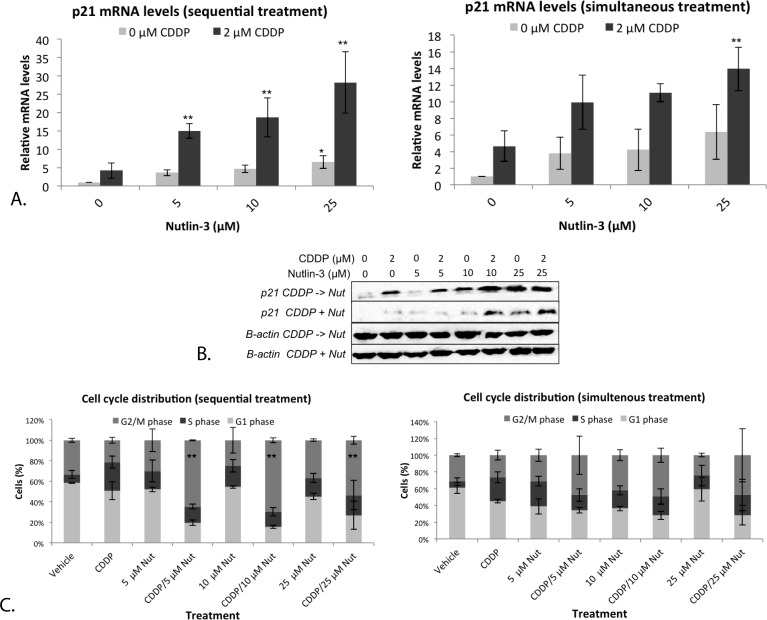
Nutlin-3 induced a strong G2/M phase arrest in combination with CDDP in the p53 wild type cell line A549 **A.** Relative mRNA expression levels of p53's transcription target p21. Cells were treated with either 2 μM CDDP; 5 μM, 10 μM or 25 μM Nutlin-3, or a sequential (CDDP –> Nutlin)/simultaneous combination therapy of both drugs for 24 hours. **B.** Corresponding p21 protein levels, β-actin was used as internal standard. **C.** Cell cycle distribution of treated cells. Cells were stained with PI and DNA content was measured by flowcytometric analysis. Cells were divided in 3 groups: G1 phase (2n); S-phase (2n-4n); and G2/M phase (4n). (*p < 0.05: significant diffence compared to 0 μM CDDP; **p < 0.05: significant difference compared to 2 μM CDDP).

P21 is an important transcription target of p53, able to induce cell cycle arrest. The mRNA levels of p21 were determined after both mono- and combination therapy. A strong and significant increase in p21 mRNA levels was observed after monotherapy with 25 μM Nutlin-3, and after sequential treatment with 5, 10 and 25 μM Nutlin-3 (Figure 6A). Again, a significant effect was not present after simultaneous treatment (Figure 6A). Similarly, the highest levels of the p21 protein were observed after sequential combination therapy (Figure 6B).

Since therapy seemed to induce a strong activation of the downstream target p21, the cell cycle distribution after both mono- and combination therapy was investigated, resulting in a very strong significant G2/M phase arrest after sequential therapy, even at low concentrations of Nutlin-3 and a markedly but not significant increase after monotherapy and simultaneous combination therapy (Figure 6C). Again, the sequential combination therapy was clearly more favorable over monotherapy or simultaneous combination therapy. Therefore, for the following experiments we focused on the sequential combination therapy.

### Duration of the cytotoxic effect

**Figure 7:**
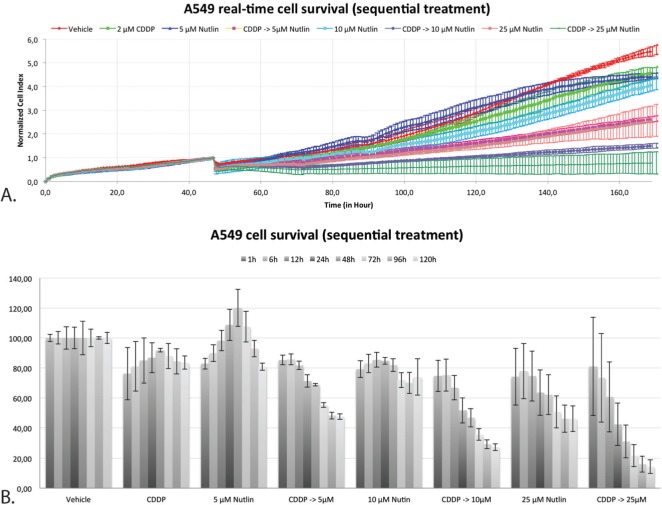
Real-time cell-viability of A549 by using the xCELLigence system after sequential treatment with CDDP and Nutlin-3 **A.** Normalized cell index over time after mono- and sequential combination therapy. **B.** Percentage of cell survival x hours after the start of treatment 2 (Nutlin-3).

By monitoring the proliferation rate of A549 in real-time using the xCELLigence system, a better insight in the duration and persistence of the cytotoxic effect after sequential treatment has been acquired. Figure 7A shows the growth curve after sequential treatment with CDDP followed by Nutlin-3. All curves were normalized at the end of treatment 1 (24h CDDP). Figure 7B shows the corresponding cell survival at 1, 6, 12, 48, 72, 96 and 120 hours after the start of treatment 2 (24h Nutlin-3). Treatment with 2 μM CDDP had only a minor effect on overall cell survival over time. On the other hand, treatment with 5 μM Nutlin-3 showed an increase of the cell index in the first 48 hours after the start of treatment, after which the number of cells gradually decreased. Sequential combination therapy showed a substantial decrease in the number of cells compared to the vehicle treated sample starting within 6 hours after treatment with Nutlin-3. After 96 hours, this decrease stagnated. These results indicate that the cytotoxic effect is clearly dependent on the addition of Nutlin-3, and is persistent over time, up to 96 hours after wash out of the drugs.

### The role of wild type p53

To determine the role of wild type p53 in the observed cytotoxic effect of the synergistic combination therapy (2 μM CDDP treatment followed by 5 μM Nutlin-3), a similar experimental setup was used for the A549 non-template control (A549-NTC), p53 deficient (A549-920) and p53 mutant cell line (CRL-5908). All cell lines were compared to the wild type p53 cell line A549.

**Figure 8:**
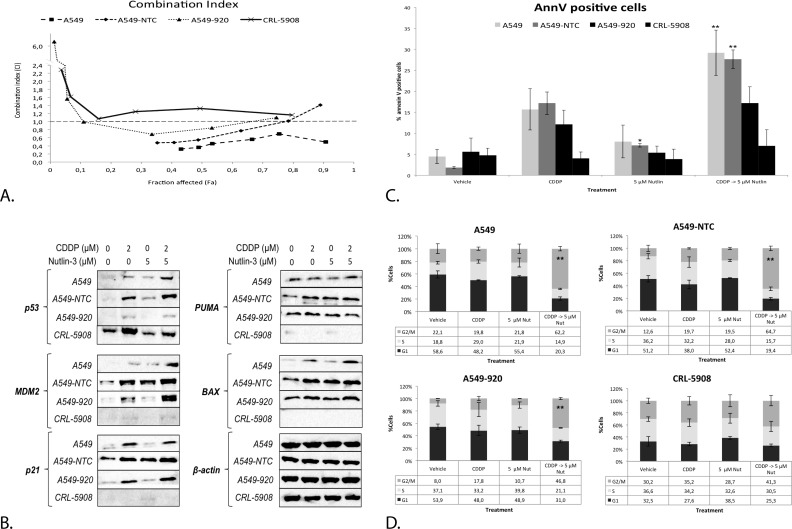
The synergistic cytotoxic effect of the sequential combination therapy was correlated with the p53 status of the cell **A.** Combination index for each CDDP concentration after sequential combination therapy in the p53 wild type cell lines A549, A549-NTC, the p53 deficient cell line A549-920 and the p53 mutant cell line CRL-5908. The supporting data for this figure (Mean IC_50_-values and mean CI) can be found in table 2. **B.** Protein expression levels of p53 and its main transcription targets MDM2, p21, PUMA, and BAX after monotherapy with CDDP or 5 μM Nutlin-3 or sequential combination therapy in each cell line. **C.** Percentage of Annexin V PerCP positive cells after treatment in all cell lines, measured by flowcytometric analysis **D.** Cell cycles distribution after treatment as previously described in all cell lines. Cells were stained with PI and DNA content was measured by flowcytometric analysis. Cells were divided in 3 groups: G1 phase (2n); S-phase (2n-4n); and G2/M phase (4n). (*p < 0.05: significant difference compared to 0 μM CDDP; **p < 0.05: significant difference compared to 2 μM CDDP).

There was no significant difference between the CDDP IC_50_-values in A549 and A549-NTC cells (IC_50_: 5.51 ± 0.72 *vs*. 4.63 ± 0.39, *p*-value = 0.066), while the p53 deficient cell line A549-920 was significantly less sensitive to CDDP (IC_50:_ 8.72 ± 0.86, *p*-value = 0.000) as for the p53 mutant cell line CRL-5908 (IC_50:_ 9.60 ± 0.63, *p*-value = 0.000) compared to A549 (Table 2). A strong to moderate synergistic effect was only observed in the p53 wild type cell line A549 and A549-NTC (CI = 0.486 ± 0.138; CI = 0.785 ± 0.370, respectively), which was strongest at low concentrations of CDDP. A549-920 was characterized by an overall antagonistic effect, but slightly synergistic at certain CDDP concentrations (CI = 1.906 ± 2.147). For CRL-5908 cells, no synergistic effect was observed at any CDDP concentration (CI = 1.453 ± 0.447). A more detailed overview of these results is given in Table 2 and Figure 8A.

**Table 2:** Overview of the IC50-values and CI obtained after sequential combination treatment for which CDDP was followed by Nutlin-3 in the p53 wild type cell lines A549 and A549-NTC, the p53 deficient cell line A549-920 and p53 mutant cell line CRL-5908

The role of p53 on cytotoxicity and synergism						
Cell line	Nutlin-3 (μM)	CDDP (0-20 μM)				
		IC_50_	StDev	*p*-value*	CI	StDev
A549	0	5.51	0.66	/	/	/
	−> 5 μM	2.67	0.26	0.003	0.486	0.138
A549-NTC	0	4.63	0.35	/	/	/
	−> 5 μM	3.69	0.27	0.024	0.785	0.370
A549-920	0	8.72	0.86	/	/	/
	−> 5 μM	9.23	1.91	0.421	1.906	2.147
CRL-5908	0	9.60	0.63	/	/	/
	−> 5 μM	9.70	1.73	0.700	1.453	0.447

The average combination index (CI) is provided for each combination therapy. CI > 1 indicates an antagonistic effect, CI = 1 an additive effect and CI < 1 a synergistic effect.

* (*p* < 0.05: significant difference in IC_50_-value compared to CDDP monotherapy)

Next, the p53 protein levels were studied. The strongest increase was observed after combination therapy in the p53 wild type cell lines. The transduced A549-920 cell line expressed some residual levels of p53 after CDDP and combination therapy, but markedly lower than the parental cell line A549 and its negative control A549-NTC. CRL-5908 showed high levels of mutant p53, which were strongest after CDDP treatment and independent of Nutlin-3 treatment (Figure 8B).

Corresponding with the p53 levels, the protein levels of p53's main transcription targets (MDM2, PUMA, BAX and p21) increased in the p53 wild type cell lines, with the most noticeable increase after combination therapy. None of these targets were observed in the p53 mutant cell line CRL-5908. As mentioned before, A549-920 cells expressed some residual p53 protein, resulting in an increased expression of MDM2 and p21 after CDDP treatment or combination therapy, but not after Nutlin-3 monotherapy. This effect in A549-920 was much less pronounced for the apoptotic related proteins PUMA, for which no increase was observed after combination therapy, and BAX, whose levels slightly increased after CDDP and combination therapy (Figure 8B). In the same way, combination therapy influenced the cell cycle distribution dependent on the p53 status of the cell. The wild type p53 cell lines A549 and A549-NTC, but also the p53 deficient cell lines A549-920 responded by a significant G2/M phase arrest. However, the arrest induced in A549-920 was significantly less than this induced in the parental cell line A549 (*p* = 0.015). The p53 mutant cell line did not show any significant changes in cell cycle distribution (Figure 8D). Finally, the induction of apoptosis was similarly dependent on the p53 status of the cell. A significant increase in apoptotic cells was only observed in the p53 wild type cell lines, but not in the p53 mutant and deficient cell line. Nevertheless, the A549-920 cell line did show an identifiable increase in apoptotic cells (Figure 8C).

## DISCUSSION

CDDP is the first line treatment for a selected NSCLC patient population administrated as platinum doublet therapy. The induction of CDDP dependent DNA damage triggers the DNA damage response activated by the ATR-Chk2 pathway resulting in p53 activation and apoptosis [18]. Tumor cells lacking functional p53 were more resistant to CDDP therapy, which was reversed upon reconstitution with wild type p53 [10]. In addition, TP53 mutations seem to negatively influence the response to CDDP therapy as a significant better overall survival and response rate was observed in TP53 wild type patients compared to TP53 mutant patients [19-21]. As the p53 pathway clearly plays an important role in the response to CDDP, the presence of adequate levels of functional wild type p53 is a necessity. By targeting the MDM2-p53 interaction in wild type p53 tumors, the p53 levels can be increased and the cytotoxic response to CDDP might be improved.

In this study, we hypothesized that the combination of CDDP with the MDM2 inhibitor Nutlin-3 could result in a synergistic cytotoxic response in p53 wild type cell lines. We focused on the sequence of administration, since Nutlin-3 is able to induce cell cycle arrest, which possibly could protect the cells from CDDP damage. Consistent with previous studies, our study showed that the response to Nutlin-3, in particular the induction of apoptotic cell death and cell cycle arrest, is p53 dependent, as only a minor cytotoxic effect was observed in the p53 deficient and mutant cell lines at high concentrations of Nutlin-3 [9, 22, 23]. Although the p53 wild type cells were sensitive to Nutlin-3 monotherapy, the apoptotic response and induction of cell cycle arrest were limited, possibly due to the lack of an activation signal of the p53 pathway, for example the induction of DNA damage by CDDP treatment.

This hypothesis was confirmed in our results indicating that the cytotoxic effect of CDDP was synergistically increased when combined with Nutlin-3. Our results are similar to those of previous studies in CDDP sensitive and resistant ovarian cancer cell lines or sarcoma cell lines, in which a low dose of CDDP was combined simultaneously with Nutlin-3 [9, 11]. We are the first to show that the sequential treatment of CDDP followed by Nutlin-3 resulted in the most potent synergistic effect compared to simultaneous treatment, both under normoxic and hypoxic conditions, in NSCLC. This effect was reflected at both the p53 protein level as well as its activity. Treatment resulted in a significant increase in p53's transcriptional targets at both mRNA and protein level and the resulting induction of G2/M cell cycle arrest and apoptotic cell death. In this study we looked at the expression levels of the pro-apoptotic proteins PUMA and BAX. PUMA localizes to the mitochondria and inhibits the anti-apoptotic proteins Bcl-2 and Bcl-X_L_, resulting in BAX activation. BAX is a transcriptional target of p53 and is able to induce mitochondrial outer membrane permeabilization, resulting in the release of cytochrome c and induction of apoptotic caspase pathway [24]. For PUMA mRNA levels, similar results were observed after simultaneous versus sequential treatment although protein levels differed. On the contrary BAX mRNA levels were only significantly increased after sequential therapy, which resulted in a strong difference in BAX protein levels, compared to simultaneous therapy. The capability of sequential treatment to induce a stronger BAX upregulation might explain the difference seen in the apoptotic response between simultaneous and sequential combination therapy.

In addition, drug dose largely affected synergism. Although combination treatment with higher doses of Nutlin-3 resulted in an increased transcription of p53 target genes and consequently increased protein levels, this did not result in a stronger synergistic effect. Adequate levels of p53 protein and its target proteins to induce their effect on cell cycle distribution or apoptosis seem to be reached at the combination of low doses. This effect was not improved by augmenting the dose of Nutlin-3 as seen in Figures 5 and 6. This could explain why the synergistic effect was strongest at low doses of CDDP and Nutlin-3.

The reduction of this response in the p53 deficient cell line, that still expressed low levels of p53, and the absence of a response in the mutant cell line indicates that this effect is strongly p53 dependent, implicating that only patients harboring wild type p53 would benefit from this combination. However, newly developed molecules like APR-246 (reactivation of mutant p53) could be able to overcome this limitation [25]. The observation that the combination therapy led to a significant G2/M phase arrest, but not to a significant increase in apoptotic cells in the transduced cell line is consistent with the view that low levels of p53 induce cell cycle arrest, whereas higher levels are needed to induce apoptosis [17]. Hence, the high levels of wild type p53 expressed after the sequential combination therapy in the parental cell line are at least partly responsible for the significant increase in apoptotic cell death compared to monotherapy.

Previous studies have also shown a p53 independent effect, likely through the inhibition of the p73-MDM2 binding or by activating E2F1 [9, 26, 27]. However, p53 independent effects only occurred at higher concentrations of Nutlin-3, which could greatly increase side effects. We did not observe a synergistic effect when combining CDDP with high concentrations of Nutlin-3 in p53 deficient/mutant cell lines (data not shown).

An important feature of newly developed therapeutics is the effect on non-malignant cells, and in general unwanted side effects in patients, especially when these new drugs are combined with commonly used chemotherapeutics [15]. Several studies have shown a cytoprotective effect of Nutlin-3 in normal cells, not only by inducing cell cycle arrest but also by blocking BAX and BAK activation in mitochondria and thereby preventing apoptotic cell death [12, 15]. We observed a similar antagonistic effect in cancer cells when administrating higher concentrations of CDDP simultaneously with Nutlin-3, but not after sequential therapy, stressing the importance to determine if the sequential combination therapy is well tolerated by normal cells *in vivo*.

Currently, several Nutlin-3 analogues like RG7112 or RG7388 are in clinical trials as monotherapy or in combination therapy [17, 28-30]. These compounds are mostly tested in sarcoma patients, eg. well-differentiated and dedifferentiated liposarcomas, because *MDM2* gene amplification occurs in about 20% of all cases, making them adequate study subjects [6, 28, 31]. However, our results show that other types of cancer, like NSCLC, could also benefit from MDM2-inhibitor combination strategies independent of the MDM2 expression status, by enhancing the expression and activation of wild type p53 in response to CDDP treatment.

Our results point to an optimal combination therapy, being the induction of DNA damage by CDDP, followed by an increase in p53 levels by Nutlin-3. A lower dose of CDDP could be used, potentially reducing side effects for NSCLC patients and improving overall prognosis. This effect was strongly dependent on the presence of wild type p53. It would be interesting to extend this research *in vivo*, comparing Nutlin-3 with newly developed MDM2 inhibitors currently in clinical development, in combination with CDDP and possibly initiate a clinical trial. The focus should be on the ideal time point for the sequential administrating of both drugs in NSCLC patients, the administrated dose and the tumors p53 status.

## MATERIALS AND METHODS

### Cell lines

The NSCLC adenocarcinoma cell lines used in this study were the parental p53 wild type A549 cell line (p53 WT, ECACC, Salisbury, England), and its isogenic derivatives A549-NTC (non-template control, p53 wild type) and A549-920 (p53 shRNA, lentiviral vector) obtained after transduction using the GIPZ lentiviral shRNA VGH5526-EG7157 viral particle set (Thermoscientific, Waltham, USA). In order to obtain a stably transduced cell line, cells were maintained in medium containing 5 μg/ml puromycin. CRL-5908 (ATCC, Rockville, USA) was used as p53 mutant cell line (R273H). Cells were cultured according to the distributor's instructions.

Cells were grown as monolayers and cultures were maintained in exponential growth in 5% C0_2_/95% air in a humidified incubator at 37°C to obtain normoxic conditions and in a humidifier Bactron IV anaerobic chamber (Shel Lab, 0% O_2_, 5% CO_2_, 95% N_2_) to obtain hypoxic conditions (<0.1% O_2_). Hypoxic conditions were initiated after first treatment. All cell lines were free from mycoplasma contamination.

### Monotherapy

Cells were plated in 96 well plates at concentrations of approximately 1800 cells/well for A549, A549-NTC, A549-920 and 2500 cells/well for CRL-5908. Cells were incubated overnight and treated for 24 hours with CDDP (0-20 μM) or Nutlin (0-50 μM) as single agents. Forty-eight hours after treatment, cell survival was determined using the sulforhodamine B (SRB) assay as previously described [32].

### Combination therapy and criteria for synergism

The combination therapies were performed in 96 well plates as described above. A549 cells were treated with CDDP (0-20 μM), combined with Nutlin-3 (5, 10, 25 μM), either simultaneous or sequential (CDDP followed by Nutlin). A549-NTC, A549-920 and CRL-5908 cells were only treated with the most optimal combination therapy. Cell survival was determined by the SRB assay. To determine the presence of a possible synergistic effect the combination index (CI) was calculated by the Chou-Talalay Method using the CalcuSyn software. A combination index CI < 1 indicates synergism, CI = 1 an additive effect and CI > 1 an antagonistic effect [33].

### XCELLigence real-time cell analysis (RTCA)

Real-time monitoring of cell viability was performed on an *xCELLigence* RTCA DP instrument (ACEA Biosciences, San Diego, USA). A detailed description of this method can be found in previously published work from our group [34]. Cells were plated in a 16-well E-plate and treated with 2 μM CDDP, and previously described Nutlin-3 concentrations. Cell viability was monitored for a period of approximately 144 hours, with kinetic measurements programmed every 15 minutes.

### RNA extraction and quantitative RT-PCR

A549 cells were plated in a 6-well plate at concentrations of 6.5 × 10^4^ cells/well. Total RNA was extracted using the TRIzol^®^ method (Life Technologies, Ghent, Belgium) after lysis. Total RNA-yield and quality were measured using the NanoDrop^®^ ND-1000 (Thermo Scientific, Erembodegem, Belgium) and stored at −80°C.

RT-PCR was performed using the Power SYBR Green RNA-to-C_T_ 1-Step Kit (Applied Biosystems, Ghent, Belgium) on the LightCycler480 (Roche, Vilvoorde, Belgium) according to the manufacturers instructions with a total of 30 ng RNA. The optimal number and type of housekeeping genes (*GAPD, RPLA13*, and *SDHA-1*) were determined using the qbasePLUS software (Biogazelle, Zwijnaarde, Belgium). Relative gene expression levels were calculated according to the comparative Ct method using the same software and plotted against the untreated sample. A panel of targets was selected based on interesting transcription targets of p53, namely PUMA and BAX (apoptosis), p21 (cell cycle arrest), MDM2 (negative feedback loop). Primers are available on request.

### Western blot

Cells were plated in a 6-well plate as described above. Cells were lysed on plates in TNN buffer. After centrifugation (5 minutes, 800rpm) the supernatants containing the isolated proteins was kept at −80°C. Protein concentrations were determined using the Pierce^®^ BCA protein assay kit (ThermoScientific). Western blot analysis was performed as described previously [35].

Following antibodies were used: rabbit monoclonal anti-p53 (1:2000, Cell Signaling Technology, Leiden, the Netherlands, no. 9282); mouse monoclonal anti-MDM2 (3G9) (1:1000, Millipore, Overijse, Belgium, no. 04-1555), rabbit monoclonal anti-p21 (1:2000, Abcam, Cambridge, UK, no. ab109199), rabbit monoclonal anti-PUMA (1:2000, Abcam no. ab33906) and rabbit monoclonal anti-BAX (1:2000, Abcam no. ab32503). Mouse monoclonal anti-β-actin was used as internal standard (1:5000, Sigma Aldrich, Diegem, Belgium). Anti-mouse and anti-rabbit HRP-labeled secondary antibodies were used (1:2000, Cell Signaling no. 7076S and no. 7074S) and chemiluminescent detection was performed using the WesternBright^TM^ Quantum Western blotting detection kit (Advansta, Temse, Belgium).

### Flow cytometry

Cells were plated in a 6-well plate as described above. Samples were analyzed using a FACScan flow cytometer (Becton Dickinson). Each sample was analyzed using 10.000 events/sample acquired. Data was analyzed using FlowJo V10. Induction of apoptotic cell death in the wild type A549 cell lines was investigated using the Annexin V-FITC apoptosis detection kit (Becton Dickinson Pharmingen, Erembodegem, Belgium) according to the manufacturers instructions. Green (Annexin V-FITC) and red (PI) fluorescence was analyzed using a FACScan flow cytometer (Becton Dickinson). Data was presented as dot plots (Annexin V plotted against PI staining). Apoptosis was determined as Annexin V positive cells, i.e. UR + LR. The number of apoptotic cells in the transduced cell lines A549-NTC and A549-920 were determined using Annexin V-PerCP-Cy^TM^5.5 (BD pharmingen) due to the interference of FITC with the transduction control protein TurboGFP. Annexin V-PerCP-Cy^TM^5.5 was also used with A549 and CRL-5908 in order to compare the results.

Cell cycle distribution was monitored according to the Vindelov method, as describe previously [36]. Data was presented as histograms of DNA content to determine cell cycle distribution (G_0_/G_1_, S and G_2_/M).

### Statistical analysis

All experiments were performed at least three times. Results, if not otherwise stated, are presented as mean ± standard deviation (SD). Statistical significance in table one and two was determined using the Mann-Whitney U test between each group and the untreated control. Statistical significance for apoptosis, cell cycle arrest and mRNA levels was determined by a two-way ANOVA test, followed by Dunnett's post hoc test for the comparison with the untreated sample or CDDP treated sample, using SPSS 22.
